# Standardization of surgical procedure for initially unresectable locally advanced pancreatic head cancer

**DOI:** 10.1007/s00423-025-03745-1

**Published:** 2025-07-17

**Authors:** Tatsuaki Sumiyoshi, Kenichiro Uemura, Ryuta Shintakuya, Kenjiro Okada, Kenta Baba, Takumi Harada, Shinya Nakamura, Koji Arihiro, Yasutaka Ishii, Shiro Oka, Shinya Takahashi

**Affiliations:** 1 Department of Surgery, Graduate School of Biomedical and Health Science, Hiroshima University, 1-2‐3 Kasumi, Minami‐ku, Hiroshima, 734‐8551 Japan; 2 Department of Gastroenterology, Graduate School of Biomedical and Health Science, Hiroshima University, Hiroshima, Japan; 3 Department of Anatomical Pathology, Hiroshima University, Hiroshima, Japan

**Keywords:** Unresectable locally advanced cancer, Pancreas head cancer, Standardization

## Abstract

**Purpose:**

The surgical procedures for initially unresectable locally advanced (URLA) pancreatic head cancer are extremely challenging owing to the severe arterial and portal vein invasions. This study aimed to propose surgical standardization plan and evaluate the efficacy of standardized procedures.

**Methods:**

Institutional conferences were held in April 2020 and March 2021 to standardize surgical procedures for URLA pancreatic head cancer. URLA cases with invasion around the celiac artery (CA) and superior mesenteric artery (SMA) were classified as UR-CA and UR-SMA types, respectively. The standardized procedures for arterial and portal venous invasion were discussed for each type, and the utility of standardization was evaluated by comparing the surgical outcomes before and after standardization (the early and late groups).

**Results:**

Five difficult surgical situations arising from arterial and portal vein invasion were identified, and the strategies for these situations were defined as standardized procedures. ‘‘Early pancreatic and splenic vein transection to the left of SMA’’ and ‘‘preresection portal vein reconstruction in cases with collateralization’’ were the two most important procedures. The early and late groups comprised seven and 11 patients, respectively. The rates of arterial resection (57.1% vs. 72.7%) and portal vein resection (PVR) (85.7% vs. 100%) were higher in the late group. Intraoperative blood loss (1650 mL vs. 530 mL, *p* = 0.001), blood transfusion rate (42.9% vs. 0%, *p* = 0.043), and severe complication rate (42.9% vs. 0%, *p* = 0.043) were significantly lower in the late group.

**Conclusions:**

Standardization of surgical procedures yielded better surgical outcomes.

## Introduction

Pancreatic ductal adenocarcinoma (PDAC) is an aggressive tumor [1], and the life expectancy of patients with unresectable PDAC is extremely poor. Two chemotherapy regimens, the oxaliplatin, irinotecan, 5-fluorouracil, and leucovorin combination chemotherapy (FOLFIRINOX) regimen, and the gemcitabine plus nab-paclitaxel (GnP) regimen have improved the survival period in this population [2–5]. Furthermore, these regimens have increased the number of patients with initially unresectable locally advanced (URLA) PDAC eligible to undergo curative-intent surgery [6–9]. Neoadjuvant chemotherapy is associated with a resection rate of 50–65% in patients with locally advanced PDAC, with a median survival rate of 15.3–31.4 months, exceeding that of palliative chemotherapy without resection [10, 11]. These results indicate the utility of conversion surgery. However, the surgical procedures for initially URLA PDAC are extremely challenging owing to the presence of arterial and portal vein invasion and cavernous transformation [12–20]. Procedures such as catheter bypass [12], mesoportal bypass [13], venous graft first approach [14], arterial-first approach [15, 16], arterial divestment [17–19], and triangle operation have yielded favorable outcomes in previous studies [20].

However, no previous study has referred to the surgical strategies in patients with both arterial and portal venous invasion. Therefore, standardized surgical procedures were discussed at institutional conferences to address these difficult situations. This study aimed to propose surgical standardization plan and evaluate its utility by comparing the surgical outcomes before and after standardization.

## Methods

### Study design

The clinical data of eligible patients were collected via a retrospective review of their medical records. This study was approved by the Institutional Review Board. The requirement for obtaining informed consent was waived owing to the retrospective study design. All procedures were performed following the tenet of the 1964 Declaration of Helsinki and its later amendments or comparable standards.

### Study population

Patients who had undergone pancreaticoduodenectomy (PD) for initially URLA pancreatic head cancer between April 2015 and May 2024 were eligible for inclusion in this study. Patients with celiac artery (CA) invasion who had undergone distal pancreatectomy with celiac axis resection were excluded. URLA PDAC was diagnosed according to the UIICC 8 th edition. The study period was set as 2015–2024 as GnP chemotherapy was covered by the National Health Insurance in December 2014 in Japan.

### Indication for surgery

Curative-intent surgery for initially URLA pancreatic head cancer was planned only when the following indications were met: (i) decrease in tumor size and the tumor exhibiting good control for ˃ 6 months under systemic chemotherapy, (ii) absence of distant metastasis, (iii) feasibility of complete tumor resection, and (iv) Hepatic artery (HA) and portal vein (PV) are reconstructible. Patients with superior mesenteric artery (SMA) stenosis are usually ineligible to undergo surgery, owing to the poor survival rates after SMA reconstruction [21].

### Basic surgical procedure

i) Pancreaticoduodenectomy.

Patients with pancreatic head cancer underwent pancreaticogastrostomy with duct-to-mucosa anastomosis and internal stent insertion [22].

ii) Arterial divestment or arterial reconstruction.

Preoperative multi-detector-row computed tomography (CT) was used to precisely evaluate arterial invasion around the CA, SMA, common hepatic artery (CHA), splenic artery (SA) and proper hepatic artery (PHA). Arterial resection and reconstruction were planned in case with arterial stenosis or irregularity. Dissection around the artery without reconstruction (arterial divestment) was considered possible in case with cancer attachment around the artery without arterial stenosis or irregularity. Metzenbaum scissors were used to perform sharp dissection while dissecting major arteries around the cancerous lesion. The dissections around the major arteries had been implemented from the distal side of these arteries before the standardization.

iii) Portal vein resection.

Portal vein resection (PVR) had been implemented at the last stage of resection before the standardization.

### Standardization of surgical procedure

Weekly conferences were held in April 2020 and February 2021 to standardize surgical procedures. Seven pancreatic surgeons attended the conferences and the difficult surgical situations and strategies were repeatedly discussed, in addition to performing a literature review.

The difficulty of standardization lies in the variety of arterial and portal vein invasions in patients with URLA PDAC. Therefore, the patients with URLA PDAC were divided into two types according to the origin of pancreatic head cancer. The UR-CA type arises from the dorsal pancreas and mainly invades the regions around CA, CHA, SA, and PHA. The UR-SMA type arises from the ventral pancreas and mainly invades around the superior mesenteric vein (SMV) and SMA. The strategies for arterial and portal venous invasions were discussed for each type, and curative-intent surgery was performed according to these standardized procedures. Patients with cancer invasion around both CA and SMA were designated into the UR-CA & SMA type, and the strategies for both UR-CA and UR-SMA were applied in such cases.

### Pathological diagnosis

The diagnosis was confirmed by two experienced pathologists specializing in biliopancreatic malignancy using surgically resected specimens.

### Outcome measures

The eligible patients were classified into two groups based on the date of standardization (the early and late groups). The early groups comprised patients who underwent curative-intent surgery between April 2015 and March 2020. The late group comprised patients who underwent curative-intent surgery between April 2021 and March 2024. The surgical outcomes and overall survival rates of the two groups were compared.

### Statistical analysis

Median values and nonparametric statistical testing procedures were used in this study. Fisher’s exact test was used to compare categorical variables, as appropriate. The Mann–Whitney U test was used to compare continuous variables. Survival curves were established using the Kaplan–Meier method, and the significance of differences was evaluated using the log-rank test*.* All statistical analyses were performed using JMP (version 13; SAS Institute, Cary, NC, USA). Statistical significance was set at a *p* value of < 0.05.

## Results

### Two representative procedures

Two most important procedures were determined among the standardized procedures.

1. Early pancreatic and Splenic vein transection to the left of SMA

Extensive cancer invasion around the CA and SMA is a serious problem. Therefore, the roots of these arteries must be taped to safely dissect between cancer and arterial adventitia. If the invasion around the CA and SMA is not severe, the mobility of the pancreatic head is maintained, and previously reported approaches such as posterior, inferior infracolic (mesenteric), left posterior, inferior supracolic (anterior), and superior approaches can be used. However, securing the roots of CA and SMA using these approaches is often difficult in cases with advanced URLA PDAC owing to poor mobility around the pancreas head. Therefore, early pancreatic and splenic vein (SV) transection to the left of SMA was used to open the window to the roots of the SMA and CA. This procedure is performed after the stomach transection at early timing of surgery.

2) Preresection portal vein reconstruction in case with severe collateralization

Portal vein resection (PVR) is usually implemented during the last stage of resection after the dissection of the dorsal cephalic plexus of pancreas. However, dissection around the pancreatic head is sometimes challenging owing to increased bleeding from well-developed collateralization and poor morbidity due to massive tumor invasion around the dorsal cephalic plexus. Preresection portal vein reconstruction is sometimes implemented prior to the dissection of the dorsal cephalic plexus after the pancreatic transection and taping of the SMA.

### Standardized procedures

#### Standardized procedures for UR-CA case

**Fig. 1:**
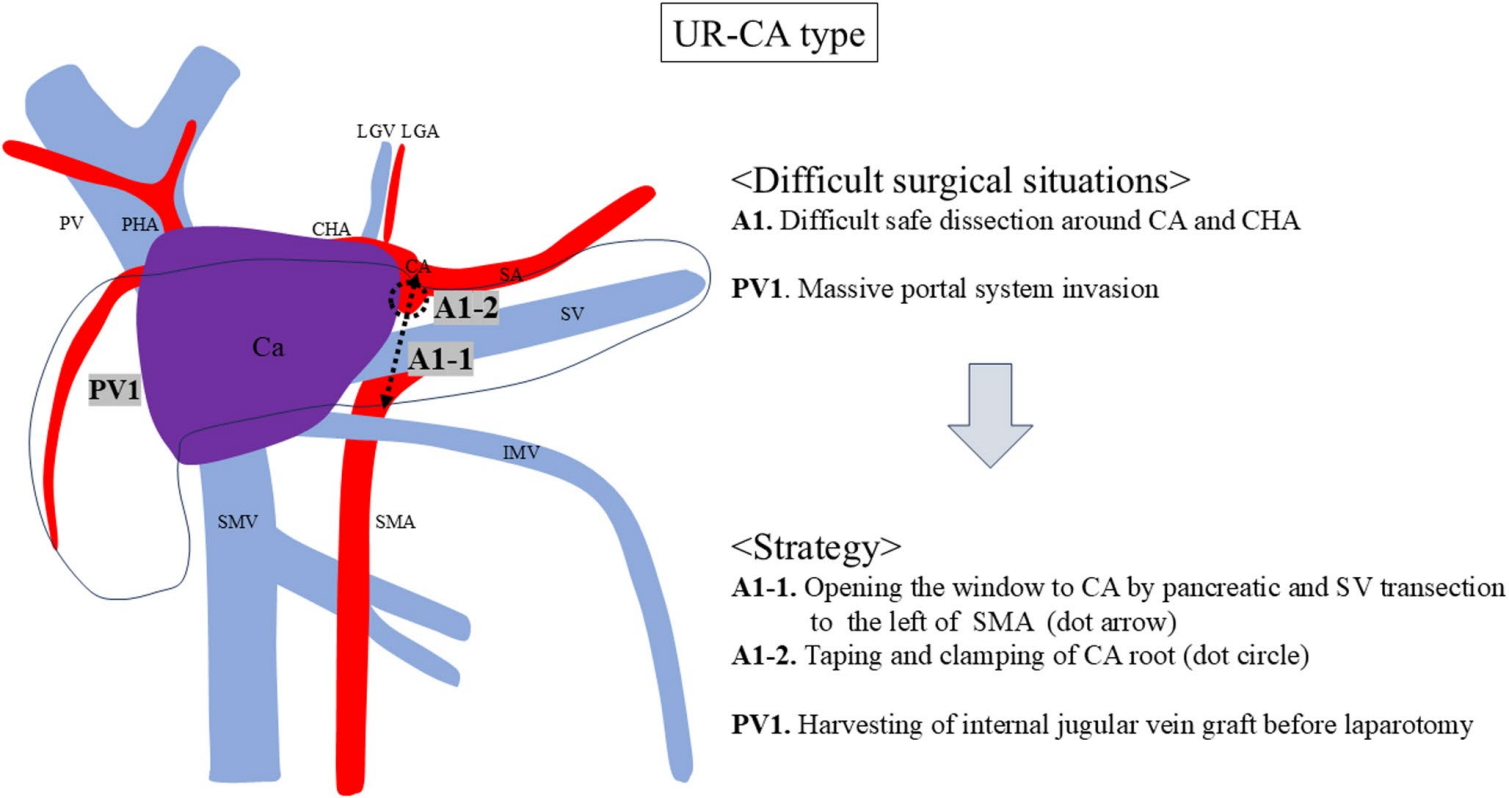
Difficult surgical situations and strategies for the UR-CA type. One difficult situations around CA (A1) and one around portal system (PV1) were identified. UR, unresectable; CA, celiac artery; CHA, common hepatic artery; PHA, proper hepatic artery; SA, splenic artery; SMA, superior mesenteric artery; HA, hepatic artery; LGA, left gastric artery; PV, portal vein; SMV, superior mesenteric vein; SV, splenic vein; IMV, inferior mesenteric vein; LGV, left gastric vein; PVR, portal vein resection; Ca, pancreatic cancer

One difficult situations around CA (A1) and one around portal system (PV1) were identified in UR-CA cases (Fig. 1). The difficult situations were as follows: difficult safe dissection around CA and CHA (A1), and massive portal system invasion (PV1).

**Fig. 2:**
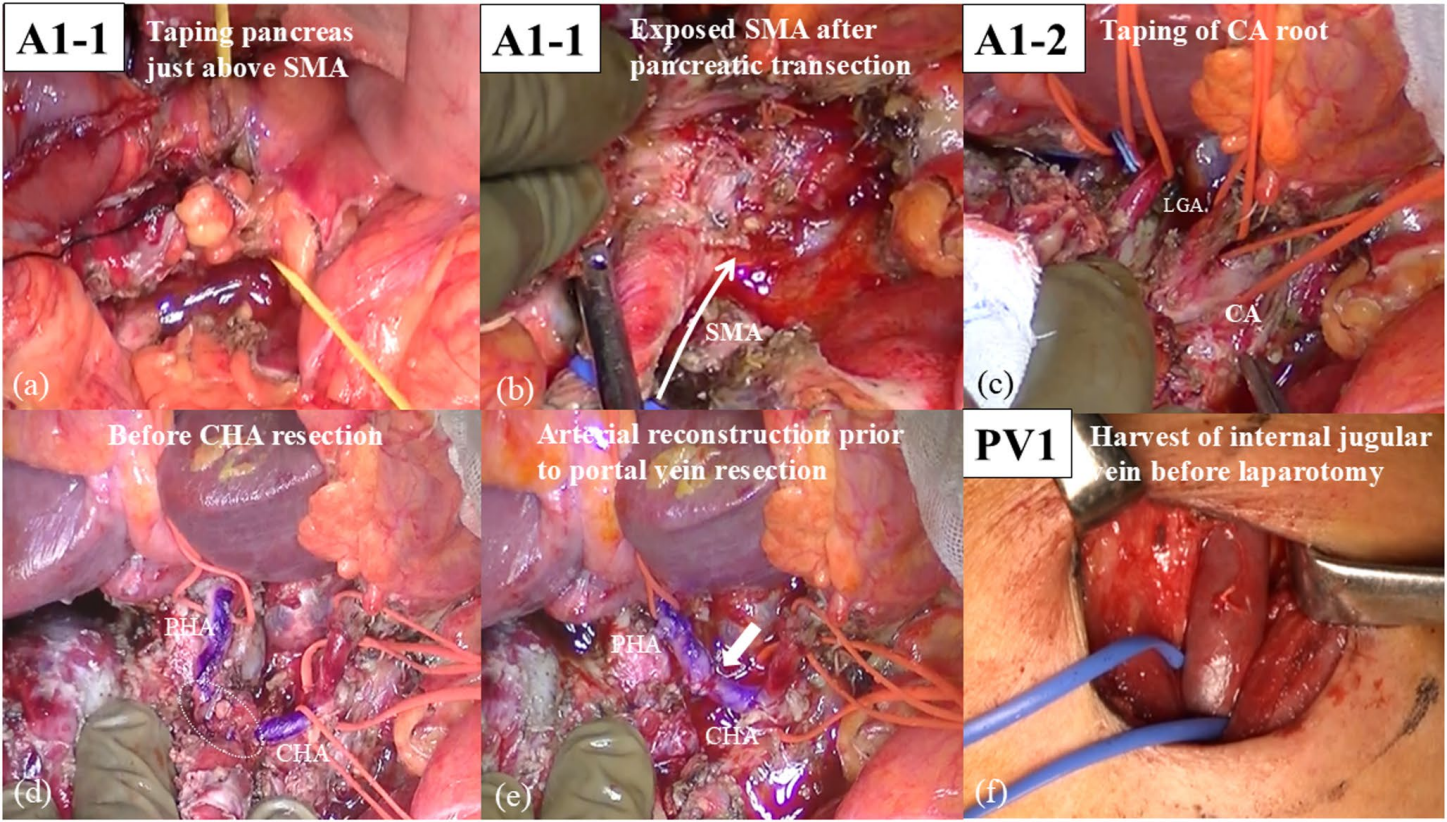
Intraoperative photographs of a UR-CA case. (**a**) The strategy for difficult safe dissection around the celiac artery (CA) and common hepatic artery is opening the window to the CA by pancreatic and splenic vein (SV) transection above or to the left of the superior mesenteric artery (SMA) (A1-1). Before pancreatic transection, the pancreatic body is taped. (**b**) The SMA is exposed after transection of the pancreas body and SV (arrow) (A1-1). (**c**) The CA root can be taped (A1-2). LGA, left gastric artery. (**d**) The strategy for hepatic infarction is performing hepatic artery reconstruction prior to portal vein resection (PVR). The left side of the hepatoduodenal ligament is dissected and the proper hepatic artery (PHA) is taped. The distal common hepatic artery (CHA) and the proximal PHA are involved by cancer (dot circle). (**e**) Hepatic arterial resection and reconstruction is performed prior to PVR to maintain hepatic blood flow (arrow). (**f**) The strategy for massive portal system invasion is harvesting the left internal jugular vein graft before laparotomy (PV1)

The strategy for difficult safe dissection around CA and CHA (A1) is ‘‘Early pancreatic and SV transection to the left of SMA’’ and it includes two steps: (i) opening the window to the CA by pancreatic and SV transection to the left of the SMA (A1-1), and (ii) taping and clamping of the CA root (A1-2). Taping the pancreatic neck is usually difficult owing to massive portal system invasion in URLA cases. Therefore, we usually transect the pancreatic body above or to the left of the SMA after the stomach transection. The pancreatic body is taped above the SMA before pancreatic transection (Fig. 2a), and the SA root is also taped, if possible, to transect pancreatic body safely. However, taping of the pancreatic body and SA root is not possible in all cases owing to the massive invasion around the CA and SA. If taping of the SA is difficult, the location of it is confirmed using intraoperative ultrasonography. Then, the pancreatic body is transected little by little to avoid injury to the SA. SV can be easily identified during pancreas transection, and SV is ligated and divided to open the window to the SMA. SV transection may not always be necessary, and however, we could not preserve it in most of our cases due to the massive cancer invasion around PV and SMV. This approach has two advantages: (i) the pancreas body can be transected above or to the left of the SMA with minimum dissection around the pancreas, and (ii) the SMA can be steadily identified after the transection of the pancreas body and SV (Fig. 2b). Subsequently, the CA root can be identified and taped after exposing the surface of abdominal aorta (Fig. 2c).

If both HA resection and PVR are necessary, surgeons should not resect them simultaneously to maintain hepatic blood flow. The following two steps are important: (i) taping of PHA in the hepatoduodenal ligament (HDL), and (ii) HA reconstruction prior to PVR. The left side of HDL is dissected and distal side of PHA is taped initially (Fig. 2d). Each of the LHA and RHA are taped in cases with broad PHA invasion. Dissection around the CHA and PHA is commenced after clamping of the CA root. Hepatic arterial resection and reconstruction are performed at this time (Fig. 2e), if hepatic artery is severely involved by the cancer. Surgeons should aim to reconstruct the HA before PVR to maintain hepatic blood flow and prevent hepatic infarction (Fig. 2e). The first candidate of arterial inflow is proximal CHA. The second candidate is SA transposition.

The strategy for massive PV system invasion comprises harvesting an internal jugular vein graft before laparotomy (PV1) (Fig. 2f). The left internal jugular vein is routinely harvested before laparotomy in cases wherein preoperative CT images show portal vein invasion longer than 3 cm. Harvesting the vein graft before laparotomy enables PV reconstruction anytime during the laparotomy.

### Standardized procedures for UR-SMA case

**Fig. 3:**
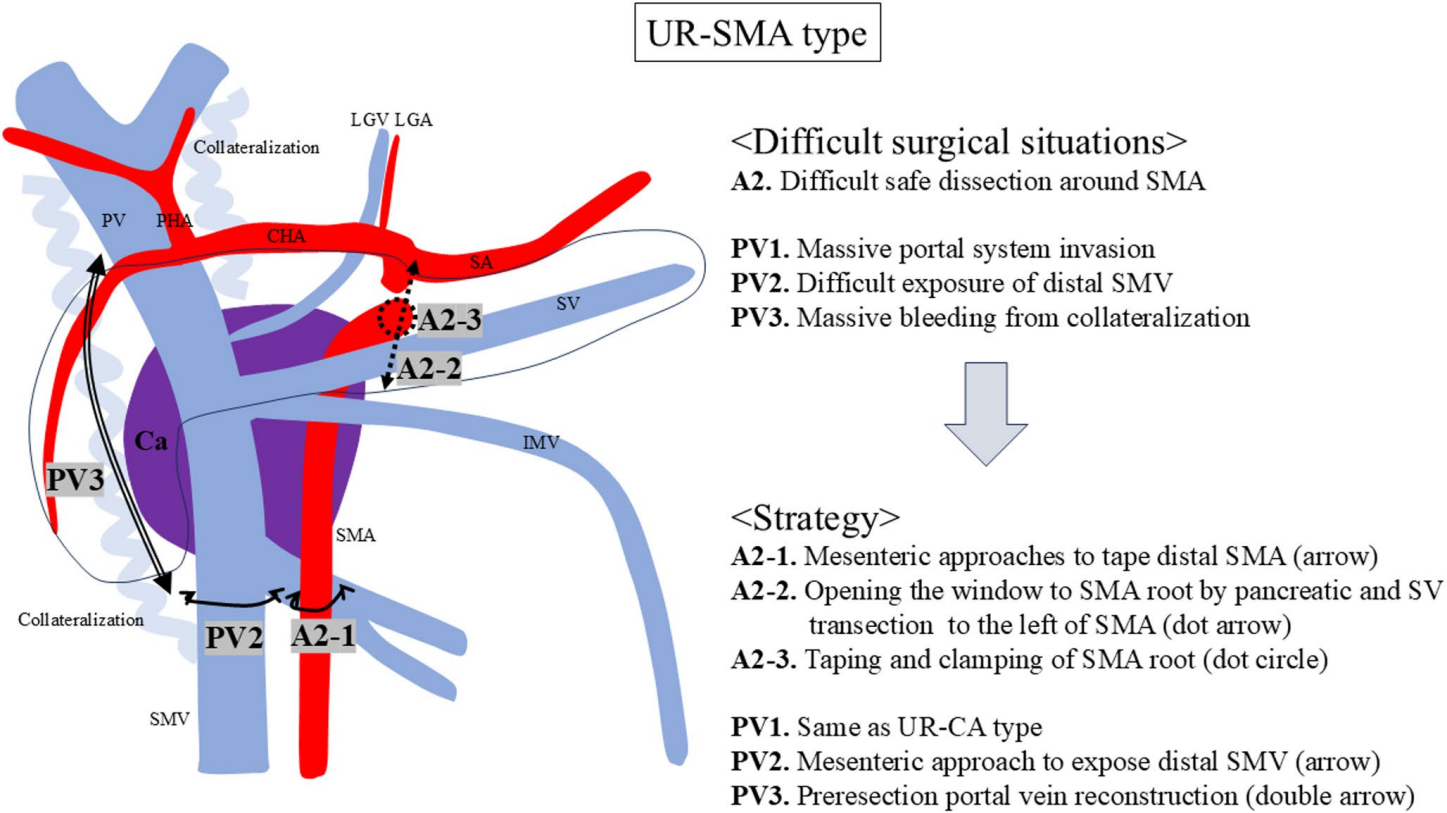
Difficult surgical situations and strategies for the UR-SMA type. One serious situation around SMA (A2) and three around portal system (PV1, PV2, PV3) were identified. UR, unresectable; CA, celiac artery; CHA, common hepatic artery; PHA, proper hepatic artery; SA, splenic artery; SMA, superior mesenteric artery; HA, hepatic artery; LGA, left gastric artery; PV, portal vein; SMV, superior mesenteric vein; SV, splenic vein; IMV, inferior mesenteric vein; LGV, left gastric vein; PVR, portal vein resection; Ca, pancreatic cancer

One difficult situation around the SMA (A2) and three around the portal system (PV1, PV2, PV3) were identified (Fig. 3). The difficult situations comprised difficult safe dissection around the SMA (A2), massive portal system invasion (PV1), difficult exposure of the distal SMV (PV2), and massive bleeding from collateralization (PV3).

The strategy for difficult safe dissection around the SMA (A2) includes the following three steps: (i) mesenteric approaches to tape the distal SMA (A2-1), (ii) opening the window to the SMA root by pancreatic and SV transection to the left of the SMA (A2-2), and (iii) taping and clamping the SMA root (A2-3).

**Fig. 4:**
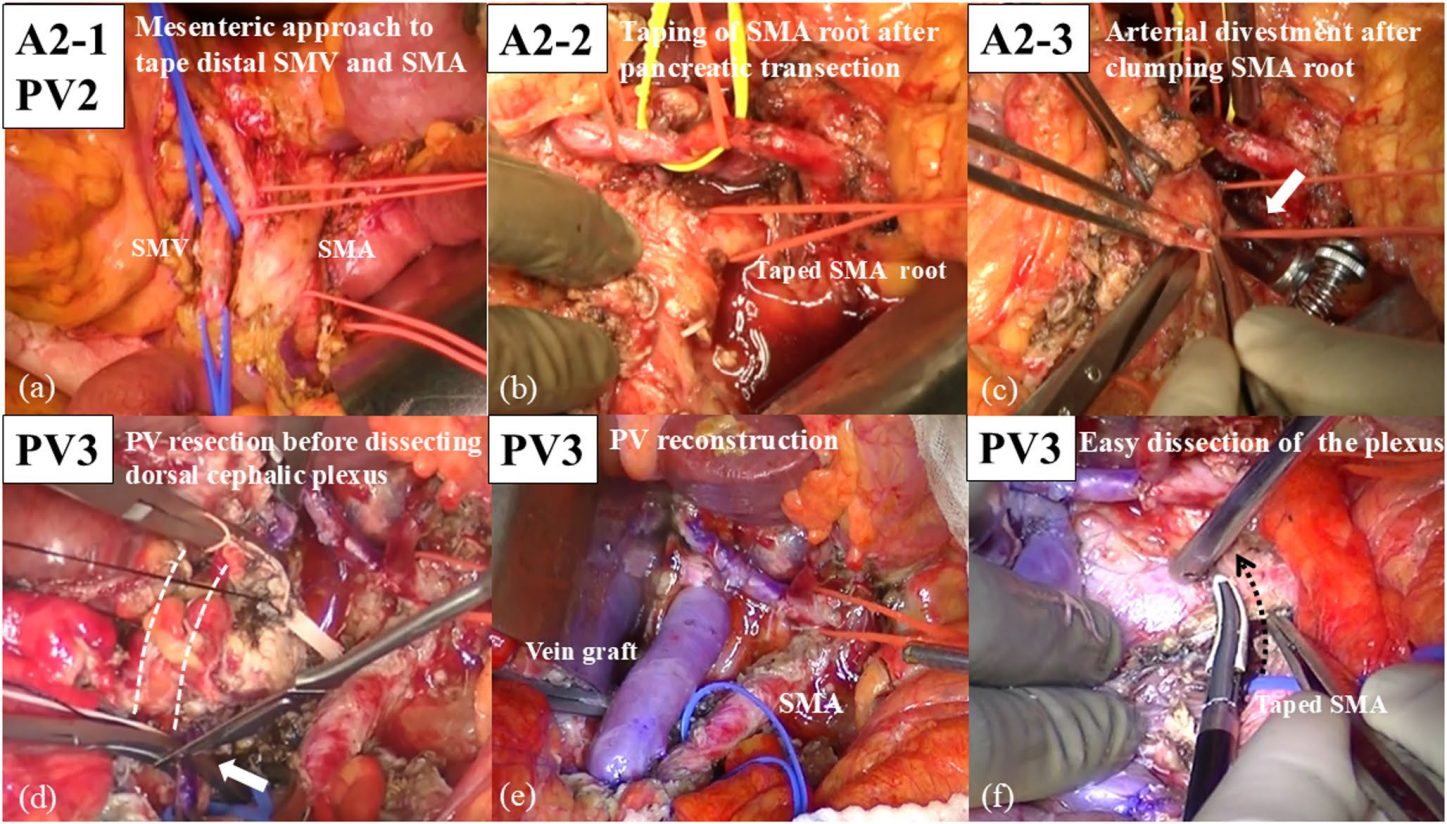
Intraoperative photographs of a UR-SMA case. (**a**) The strategy for difficult dissection around the distal superior mesenteric artery (SMA) and distal superior mesenteric vein (SMV) is the mesenteric approach (A2-1, PV2). This approach is optimal for securing the distal SMA and SMV in case of massive invasion around those vessels. The infracolic transverse mesocolon is dissected and the SMA and SMV are taped. (**b**) The window to the SMA root is opened by pancreatic and splenic vein transection above or to the left of the SMA (A2-2), and the SMA root is taped. (**c**) Arterial divestment around the SMA after clamping the SMA root (A2-3) (arrow). Metzenbaum scissors are used to perform sharp dissection. (**d**) The strategy for massive bleeding from cavernous transformation is preresection portal vein (PV) reconstruction before specimen extraction (PV3). The dotted line indicates that the PV and SMV are involved by cancer. PV resection is implemented before dissecting the dorsal cephalic plexus of pancreas. The arrow indicates the cutting of distal SMV by knife. (**e**) Preresection PV reconstruction using left internal jugular vein graft before dissecting the cephalic plexus of pancreas. (**f**) The cephalic plexus of pancreas can be easily dissected after early PV reconstruction (dotted arrow)

Firstly, the mesenteric (inferior infracolic) approach is optimal for securing the distal SMA and distal SMV (A2-1) (Fig. 4a). In UR-SMA cases originating from ventral pancreas, the invasion around the SMV is often more severe than that observed in UR-CA cases. The anterior lobe of transverse mesocolon is often involved, making it difficult to expose the SMA by supracolic approach. The distal SMA can be exposed and taped steadily using the mesenteric approach. The next step is ‘‘pancreatic and SV transection to the left of SMA’’, as well as UR-CA cases (A2-2). The SMA root can be easily exposed by this procedure. It seems paradoxical that pancreatic transection above SMA is possible in case with severe invasion around the SMA. However, the cancer usually originates from the ventral pancreas in UR-SMA cases, and the invasion around SMA comes from dorsal retroperitoneum. Therefore, pancreatic transection above the SMA is usually possible. Thirdly, cancer invasion around the SMA can be safely dissected by clumping of the SMA root (A2-3) (Fig. 4b and c). The distal SMA is also clamped if the bleeding around the SMA is uncontrollable.

The strategy for massive PV and SMV invasion (PV1) is the same as described in standardized procedures for UR-CA case. The strategy for difficult exposure of the distal SMV (PV2) is the mesenteric approach, as well as the distal SMA. The invasion around SMV in UR-SMA case is generally more severe than that in UR-CA case. The mesenteric approach enables the accurate identification and safe dissection around the distal SMV (Fig. 4a).

The strategy for massive bleeding from collateralization (PV3) is ‘‘Preresection portal vein reconstruction’’ prior to specimen extraction. After the transection of pancreatic body and SV, the hepatic side of PV is taped. Both PV and SMV are dissected to the maximum extent and cut just around the cancer-invaded portion (Fig. 4d). The harvested internal jugular vein graft is interposed between PV and SMV, and PV reconstruction is performed using end to end anastomoses (Fig. 4e). This preresection portal vein reconstruction dramatically improves both the bleeding tendency from collateralization and poor mobility around the pancreas head. The dorsal cephalic plexus of pancreas can be cut easily after this procedure (Fig. 4f).

### Standardized procedure for UR-CA & SMA case

**Fig. 5:**
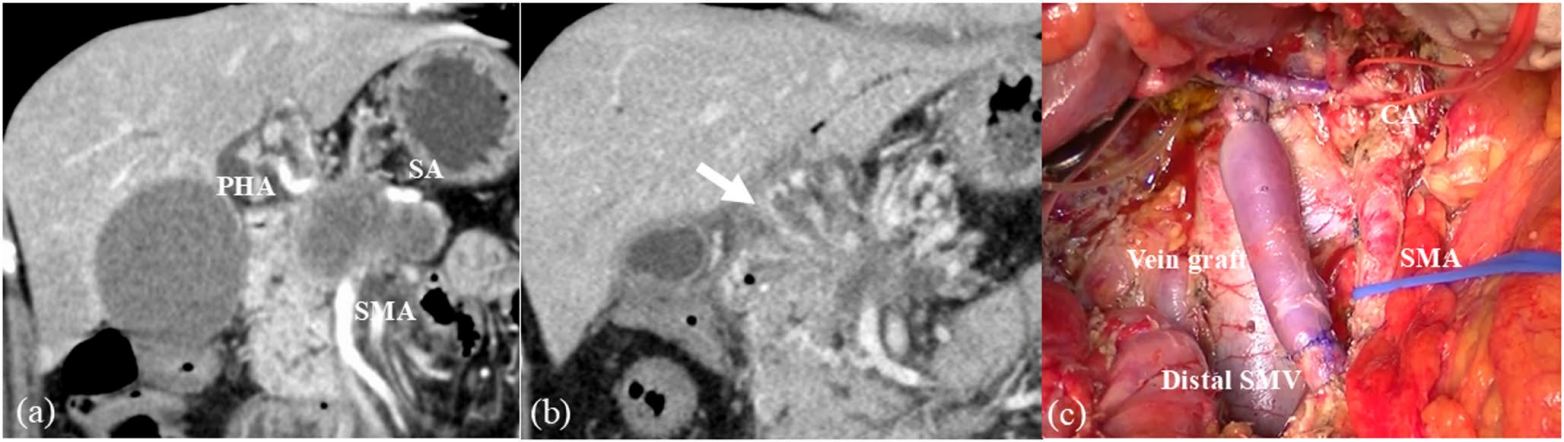
A representative UR-CA & SMA case. (**a**) Computed tomography shows remarkable cancer invasions around the celiac artery, common hepatic artery, proper hepatic artery (PHA), splenic artery (SA), superior mesenteric artery (SMA), portal vein and superior mesenteric vein. (**b**) The tumor shrank after the preoperative chemotherapy. However, remarkable collateralization around the pancreas head emerged owing to the occlusion of the portal vein and superior mesenteric vein (arrow). (**c**) The standardized procedures for the UR-CA case and UR-SMA case were utilized and the patient received pancreaticoduodenectomy with common and proper hepatic arteries resection & reconstruction, splenic artery resection, and portal vein resection & reconstruction using left internal jugular vein graft. Her postoperative course was uneventful, and she remained recurrence-free for postoperative three years

UR-CA & SMA case is the most intractable URLA case. However, utilizing both procedures for UR-CA and UR-SMA cases facilitates safe surgery. Figure 5 represents a successfully treated UR-CA & SMA case.

### Surgical outcomes

#### Eligible patients

Seven patients with initially URLA pancreas head cancer underwent curative-intent surgery during the 5 years before standardization (early group), and 11 patients underwent surgery during 3 years after standardization (late group).

#### Comparison of preoperative outcomes between the early and late groups

No significant differences were observed between the groups in terms of age, body mass index, sex, type of URLA, and preoperative treatment (Table 1). The rate of UR-CA & SMA cases was greater in the late group (14.3% vs. 27.3%).

**Table 1:** Preoperative outcomes in early and late groups

Characteristics No. (%) or median (range)	Early group (*n *= 7)	Late group (*n *= 11)	*P* value
Age	64 (58–77)	65 (48–81)	0.785
BMI (kg/m^2^)	19.2 (16.9–31.3)	23.3 (18.5–26.0)	0.103
Sex (male)	6 (85.7)	8 (63.6)	0.308
Type of URLA: UR-CA/UR-SMA/UR-CA & SMA	3 (42.9)/3 (42.9)/1 (14.3)	6 (54.5)/2 (18.2)/3 (27.3)	0.512
Preoperative treatment			0.171
Gemcitabine based chemotherapy	6 (85.7)	6 (54.5)	
FOLFIRINOX	1 (14.3)	5 (45.5)	

*BMI* body mass index, *URLA* unresectable locally advanced, *CA* celiac artery, *SMA* superior mesenteric artery, *FOLFIRINOX* 5-fluorouracil, leucovorin, irinotecan, and oxaliplatin

#### Comparison of surgery-related findings between the early and late groups

The most commonly performed procedure was PD in both groups (57.1% vs. 81.8%, respectively) (Table 1). All rates of arterial resection (57.1% vs. 72.7%), portal vein resection (85.7% vs. 100%), arterial and portal vein resection (42.9% vs. 72.7%), and SV reconstruction (0% vs. 18.2%) were higher in the late group, although the differences were not statistically significant. The resected arteries were hepatic artery or splenic artery in both groups. No patient in the early group underwent portal vein reconstruction using graft interposition; in contrast, all patients in the late group underwent this procedure (*P* < 0.001). The operation time in the late group was significantly longer owing to the higher rate of vascular resection and reconstruction (median time: 504 min vs. 665 min, *P* = 0.008). However, intraoperative blood loss (1650 ml vs. 530 ml, *p* = 0.001), blood transfusion rate (42.9% vs. 0%, *p* = 0.043), severe complication rate with Clavien-Dindo grade *>* 3 (42.9% vs. 0%, *p* = 0.043), and vascular complication rate (42.9% vs. 0%, *p* = 0.043) were significantly lower in the late group.

#### Comparison of pathological findings between the early and late groups

No significant differences were observed between the groups in terms of the UICC T factor and N factor. The R0 resection rates were 85.7% and 90.9% in the early and late group, respectively (*p* = 0.732) (Table 2).

**Table 2:** Postoperative outcomes in early and late groups

Characteristics No. (%) or median (range)	Early group (*n* = 7)	Late group (*n* = 11)	*P* value
< Surgery-related findings>			
Surgery (PPPD/PD)	3 (42.9)/4 (57.1)	2 (18.2)/9 (81.8)	0.255
Arterial resection	4 (57.1)	8 (72.7)	0.627
Hepatic artery resection	3 (42.9)	7 (63.6)	0.387
Splenic artery resection	1 (14.3)	1 (9.1)	0.732
Portal vein resection	6 (85.7)	11 (100)	0.389
Graft interposition	0 (0)	11 (100)	< 0.001
Arterial and portal vein resections	3 (42.9)	8 (72.7)	0.205
SV reconstruction*	0 (0)	2 (18.2)	0.232
Operation time (min)	504 (386–640)	665 (523–948)	0.008
Blood loss (ml)	1650 (1280–5295)	530 (82–1487)	0.001
Blood transfusion	3 (42.9)	0 (0)	0.043
Complication (grade > 3^a^)	3 (42.9)	0 (0)	0.043
Vascular complication Hepatic artery occlusion Portal vein stenosis	3 (42.9) 2^#^ (28.6) 1 (14.3)	0 (0) 0 (0) 0 (0)	0.043
CRPF	1 (14.3)	0 (0)	0.389
In-hospital death	0 (0)	0 (0)	NA
*Postoperative hospital stay (day)*	*24 (18–36)*	*22 (15–35)*	*0.566*
< Pathological findings>			
UICC T stage (T3 or T4)	2 (28.6)	2 (18.2)	0.601
UICC N stage (N0/N1/N2)	0 (0)/5 (71.4)/2 (28.6)	5 (45.5)/4 (36.4)/2 (18.2)	0.109
Residual tumor R0/R1	6 (85.7)/1 (14.3)	10 (90.9)/1 (9.1)	0.732

*PPPD* pylorus-preserving pancreaticoduodenectomy, *PD* pancreaticoduodenectomy, *SV* reconstruction*, splenic vein reconstruction using splenic vein-left renal vein anastomosis; Grade > 3^a^, Clavien-Dindo grade *>* 3; 2^#^, The two patients did not show hepatic abscesses owing to the well-developed inferior phrenic collaterals; *CRPF* clinically relevant pancreatic fistula, *UICC* Union for International Cancer Control

#### Survival period in the early and late groups

**Fig. 6:**
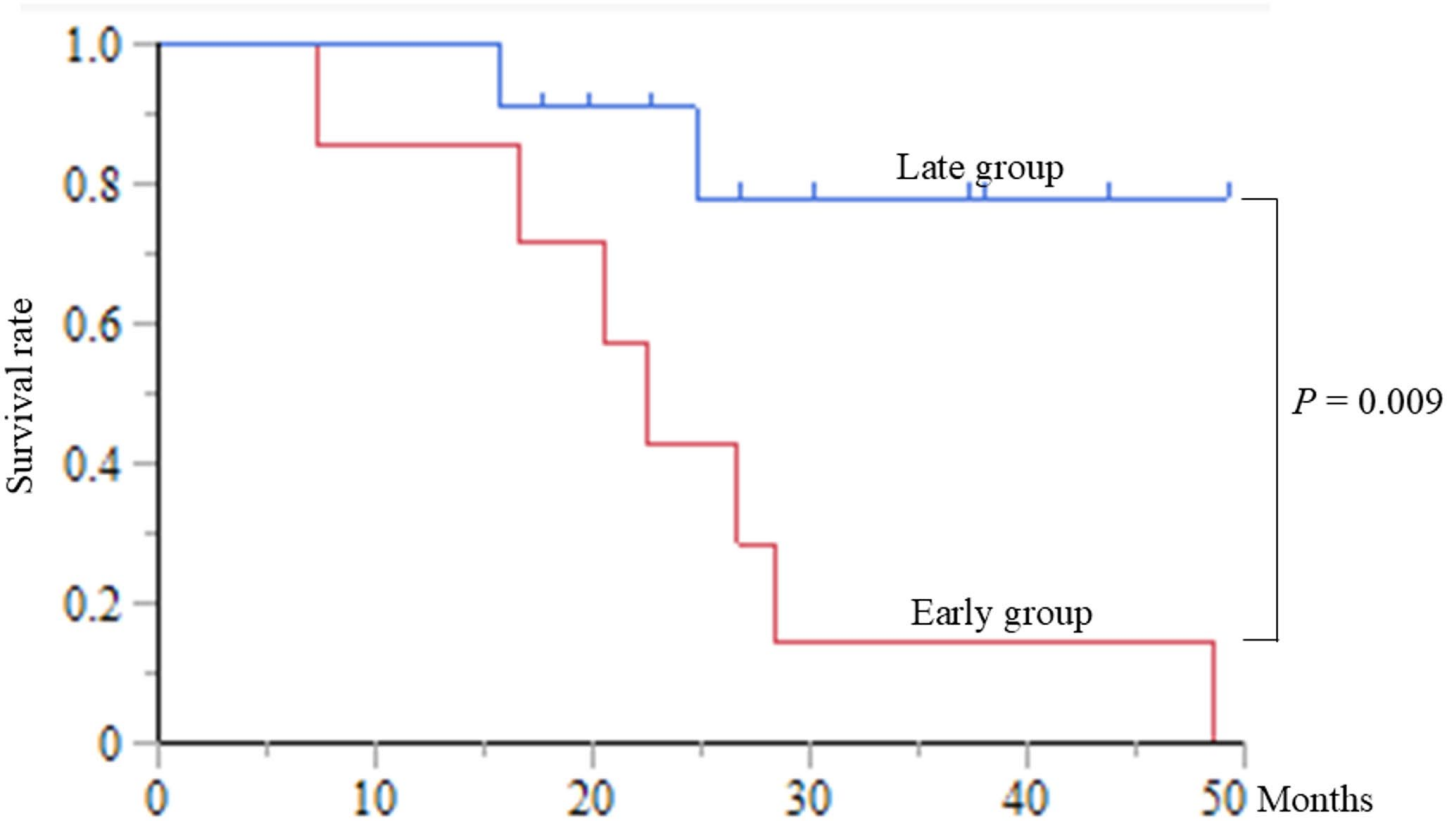
Overall survival curves for the early and late groups

The median survival was 22.5 months and not reached in the early and late groups, respectively (*P* = 0.009) (Fig. 6)*. *The 1-, 2-, and 3-year survival rates were 85.7%, 42.9%, and 14.3%, respectively, in the early group and 100%, 90.9%, and 77.9%, respectively, in the late group (Fig. 6)*.*

## Discussion

Surgery for URLA PDAC is extremely difficult as both arterial and portal venous resections are often required under poor mobility around the pancreas, and occasionally under intractable bleeding tendency from cavernous transformation. More than 70% of patients in the late group underwent both arterial and portal vein resections at our institution. Some previous studies have proposed effective strategies against arterial or PV invasions [12–20]. However, no studies have developed any strategies for cases with both arterial and PV invasions as the location and extent of vascular invasion vary depending on the tumor location and its size. Therefore, the URLA cases were classified into two types according to the origin of cancer (UR-CA and UR-SMA) in the current study, and standardized procedures were determined in each type.

In case with arterial invasion, securing the arterial root is deemed the most important step, regardless of the type of URLA. Subsequent dissections around the CA or SMA can be performed safely if their roots can be secured. However, CA and SMA are originally located on the back of the pancreas, and extensive invasion around the CA and SMA is observed in patients with URLA, which hinders the approach to these roots. Several artery-first approaches have been proposed;^16^ however, taping of the CA and SMA roots using these approaches is difficult in patients with URLA PDAC. The dorsal approach with Kocher mobilization seems to be optimal for approaching the SMA root directly; however, cancer invades the retroperitoneal space widely, and the poor mobility of the pancreas head hinders securing the SMA root in many URLA cases. The inferior supracolic (anterior) approach is also sometimes unsuitable for securing the SMA root owing to massive invasion around the anterior lobe of mesocolon. The superior approach from the cranial side is also unsuitable for securing the CA root owing to the massive invasion around the CA. Regardless the approach selected, the CA or SMA roots can be taped only after risky dissection around the distal sides of these arteries. Therefore, ‘‘Early pancreatic and SV transection to the left of SMA’’ was devised by the authors to tape the arterial roots firstly. Taping of the SMA and CA roots could be achieved by using this technique with less amount of bleeding in all cases after the standardization. The biggest advantage of this technique is the direct approach to the SMA and CA roots, and it allows for subsequent safe dissection around these arteries by clamping their roots. The disadvantage of this technique is that pancreatic transection must be performed before judging the presence or absence of cancer invasion around the CA and SMA roots. However, this judgement can be performed only after exposing the CA and SMA roots. Thus, preoperative estimation of cancer invasion around the CA and SMA using CT images is important. None of the patients were intraoperatively diagnosed as unresectable after pancreatic transection, and the R0 resection rate was high at 90.9%.

Regarding PV invasion, extensive long involvement is often observed. Fujii et al. reported that the length of SMV/PV resection exceeding 31 mm was an independent predictor of medium-term severe anastomotic stenosis [23]. Therefore, left internal jugular vein grafts were routinely harvested before laparotomy in cases with longer PV involvement, and PV stenosis was not observed in any of the patients after the standardization. The left renal vein (LRV) grafts are not used usually considering the possibility of SV reconstruction. SV reconstruction was planned when obvious gastric venous congestion was observed after the pancreatectomy, and SV-LRV anastomosis was implemented*.*

Similarly, the iliac vein is not used because it may be used as the access route of the port-systemic shunt to decrease portal pressure. Some previous reports have described the long PVR and direct anastomosis without graft interposition after liver mobilization and Cattell-Braasch Maneuver [24–26]. However, the length of PVR can be very long in URLA cases, and even Cattell-Braasch Maneuver can be difficult to perform owing to cavernous transformation.

Another problem of massive PV invasion observed in UR-SMA case is cavernous transformation. Nakao et al. reported the utility of catheter bypass for portal pressure reduction [12]. Schmidt et al. reported the utility of venous bypass graft first [14], and the distal SMV and PV are bypassed using artificial blood vessel immediately after laparotomy. Graft resection and re-anastomosis must be performed to shorten the artificial graft after specimen extraction in this procedure. Further, John’s Hopkins University group reported the utility of mesocaval shunt [13]. This procedure may be especially useful, when surgeons cannot approach PV in hepaticoduodenal ligament owing to the developed cavernous transformation. Meanwhile, our preresection portal vein reconstruction is performed after pancreatic transection and before dissection of the cephalic plexus of pancreas. The biggest advantage of this procedure is the simpleness. The catheter bypass and mesocaval shunt require the revascularization outside of resection area, and however, “Preresection portal vein reconstruction” does not require other revascularization*.* By completing portal vein reconstruction before dissection of the plexus, dissection around the pancreatic head become easier and the risk of bleeding is remarkably decreased owing to the reduced PV pressure.

The excellent surgical outcomes achieved after standardization indicate the clinical utility of these standardized procedures. The rates of both arterial and portal vein resections were almost twice in the late group, and however, all cases were transfusion-free and had no serious complications after the surgeries.

This study has certain limitations. Firstly, this was a single-center study with a small sample size. Therefore, the standardization proposed herein may not be the optimal solution. The other type of URLA cases such as massive PV invasion without arterial invasion can exist*.* However, this is the first that described the both strategies for the arterial and PV invasions, and the strategies can be one option to get through the difficult surgery. Second, our strategies were counterplans for the most advanced URLA cases with arteriovenous involvement and cavernous transformation; thus, they may be unnecessary in some URLA cases. Third, the influence of the building up of surgical team experiences on surgical outcome cannot be absolutely excluded, although the preoperative management and basic surgical techniques are same in the early and late groups*.*

In conclusion, the standardization of surgical procedures yielded better surgical outcomes in patients with initially URLA pancreatic head cancer. 

## Data Availability

No datasets were generated or analysed during the current study.
